# DOES BECOMING FRAIL MEAN THE END? SOCIAL, LIFESTYLE, AND HEALTH SURROGATES OF RESILIENCE IN THE MOST VULNERABLE

**DOI:** 10.1093/geroni/igad104.1447

**Published:** 2023-12-21

**Authors:** Chenkai Wu, Qianli Xue

## Abstract

Frailty is a prevalent geriatric syndrome characterized by reduced reserve to stressors and increased vulnerability to adverse outcomes. Despite many efforts, reversing the frail status remains difficult, primarily due to a lack of understanding of its etiology. Intervention efforts have primarily focused on improving functions and alleviating symptoms instead of targeting frailty-causing biology and treating the syndrome. Although no magic pill exists for treating frailty, becoming frail doesn’t mean the end of life. Frail individuals are a socioeconomically, behaviorally, and clinically diverse group. Identifying socioeconomic, lifestyle, and health features that could mitigate the frailty-induced health risks and promote resilience is critical for guiding patient-centered management of frailty. This symposium has a collection of four studies focusing on different ways to promote health and reduce risks among the most vulnerable. The first study suggested that adherence to a healthy lifestyle may provide an excellent opportunity to decrease the risk of adverse outcomes among the frail, especially those living in an unfavorable social environment. The second study found frailty to be associated with higher healthcare expenditure only among persons with poor self-rated health, suggesting individuals reporting good health are resilient to frailty-induced health risks. The third study found that physical frailty and self-rated health were overlapping but distinct constructs. Good self-rated health was predictive of low mortality among frail individuals, and the association was stronger among the frail than the non-frail. In the fourth study, intrinsic capacity, assessed by a validated, multidimensional tool, provided prognostic value for healthcare utilization beyond multi-morbidity.

